# Organisation and content of supervised group exercise for people with axial spondyloarthritis in The Netherlands

**DOI:** 10.1007/s00296-020-04742-x

**Published:** 2020-11-26

**Authors:** Bas Hilberdink, Florus van der Giesen, Thea Vliet Vlieland, Salima van Weely

**Affiliations:** 1 Department of Orthopaedics, Rehabilitation and Physical Therapy, Leiden University Medical Center, j11, P.O. Box 9600, 2300 RC Leiden, The Netherlands; 2 Department of Rheumatology, Leiden University Medical Center, Leiden, The Netherlands

**Keywords:** Axial spondyloarthritis, Group exercise, Supervision, Organisation, Content, Survey

## Abstract

Supervised group exercise (SGE) is recommended for people with axial spondyloarthritis (axSpA). Recent literature suggests that its contents and dosage must probably be revised. As a first step towards renewal, this study examined the current SGE organisation and content for people with axSpA in The Netherlands. A pen-and-paper survey was sent to the boards of the 82 local patient associations affiliated with the Dutch Arthritis Society in 2016. One member of each board was asked to complete questions on the nature and organisation of SGE and one of the supervising therapists to complete questions on the SGE supervision and contents. The questionnaire was returned by representatives of 67/82 (82%) local patient associations, of which 17 (25%) provided axSpA-specific SGE (16/17 SGE programmes with both land-based exercise and hydrotherapy and 1/17 with only hydrotherapy). These involved in total 56 groups with 684 participants and 59 supervisors, of whom 54 were physical therapists and 21 had had postgraduate education on rheumatic and musculoskeletal diseases (RMDs). Besides mobility and strengthening exercises and sports (17/17), most programmes included aerobic exercise (10/17), but rarely with heart rate monitoring (1/17), patient education (8/17), periodic assessments (2/17), or exercise personalisation (1/17). In the Netherlands, a quarter of local patient associations organised axSpA-specific SGE, mostly containing land-based exercises combined with sports and hydrotherapy. Most supervisors lacked postgraduate education on RMDs and most programmes lacked intensity monitoring, patient education, periodic assessments, and personalisation, which are needed for optimising exercise programmes according to current scientific insights.

## Introduction

Axial spondyloarthritis (axSpA) is an inflammatory rheumatic disease that primarily affects the spine and sacroiliac joints, and is characterised by chronic back pain and stiffness that often decreases with exercise [1, 2]. Exercise is proven effective in reducing symptoms and increasing spinal mobility, cardiorespiratory fitness, and physical functioning of people with axSpA [3–10]. The literature in particular demonstrates that supervised group exercise (SGE) is more effective in improving quality of life, spinal mobility, and patient global assessment than unsupervised, individual exercise [5, 10–13]. However, it must be noted that the evidence supporting SGE in AxSpA is mostly based on studies that were published quite some time ago and the main focus of the interventions in these trials concerned joint mobility exercises [10, 14]. This contrasts with recent literature, suggesting that SGE for people with axSpA would ideally also include patient education and muscle strengthening and aerobic exercises, with the right frequency and intensity, that are personalised according to regular reassessments [6–11, 15, 16].

The implementation of these new insights in current practice of SGE in axSpA, however, appears to be insufficient. In the Netherlands, the nature and contents of many of the SGE programmes for axSpA patients are still based on an intervention used in a randomised, controlled trial from a few decades ago [17]. In that period, an inventory of practice was made, finding that there were 31 SGE classes in the Netherlands, which all used land-based joint mobility and muscle strengthening exercises (100%), often combined with sports (84%) and hydrotherapy (72%) [18]. The SGE classes took place weekly, with an average duration of 95 min (range 50 and 155). Furthermore, the large majority of the supervisors were physical therapists (90%) and only few had had postgraduate education on SGE (8%) [18]. A recent study in four regions in the Netherlands where SGE for patients with axSpA is provided showed that current practice appears similar to the situation in 1991 [19]. That study suggests that SGE contents and dosage must be revised to meet current scientific insights. Additional knowledge regarding current SGE engagement, organisation, and content among people with axSpA from other studies appears to be scarce. Two Swiss studies report that 68 axSpA-specific SGE groups are organised by the Ankylosing Spondylitis Association of Switzerland, in which SGE is provided mostly on land, often complemented with hydrotherapy, on a weekly basis, supervised by a physical therapist and focusing on muscular strength and joint mobility [20, 21]. In the United Kingdom, it is reported that land-based SGE and hydrotherapy are organised by 74 and 65 branches from the National Axial Spondyloarthritis Society (NASS), respectively [22]. No information is provided on the specific contents or organisational characteristics of SGE for people with axSpA. Regarding overall SGE use, a recently published cross-sectional study on the engagement of people with axSpA in SGE found that, in the Netherlands and Switzerland, 9% and 30% of the respondents are attending SGE, respectively, and that these numbers are declining over time, while the average age of SGE participants is increasing [23].

Since detailed information on the actual provision of SGE for axSpA on the national level is missing, this study examines the current use, content, supervision, and organisational characteristics of SGE for axSpA patients in the Netherlands. This is a first step towards a revision of the content and dosage of SGE; a development which appears to be supported not only form a scientific viewpoint but from the patients’ perspective as well [19].

## Methods

### Design

This cross-sectional study, conducted in 2016, constituted the basis for a follow-up project aiming to improve SGE for people with axSpA. The first step concerned a pilot implementation project including four local patient associations [19]. A survey was sent to the boards of 82 local patient associations affiliated with the Dutch Arthritis Society in the Netherlands at that time. The medical ethics committee of the Leiden University Medical Center approved the study protocol and judged that a full review was not needed due to the observational nature of the study and that subjects who were invited were free to complete the survey or not (CME file P14.326). The study was conducted in line with the national and international regulations regarding the handling of personal data in research [24].

### Subjects

At the time of sending the survey in 2016, 82 local patient associations were affiliated with the Dutch Arthritis Society: some of these associations are axSpA-specific and some are for patients with any rheumatic and musculoskeletal disease (RMD), including axSpA [25]. All associations organising SGE for people with axSpA, regardless of whether the SGE was exclusively for people with axSpA or not, were included in the present study.

### Assessments

A pen-and-paper survey was sent to the boards of the patient associations accompanied by an invitational letter, signed by the Patient Interests department of the Dutch Arthritis Society. This questionnaire approach was chosen, as the Dutch Arthritis Society was involved in the distribution and collection of the surveys and this was well in line with their usual way of communication with the local patient associations. The survey contained two parts: one part to be completed by a board representative and the second part by a supervisor of SGE, but only in case SGE was provided (either axSpA-specific or for any RMD). The survey was self-developed and used dichotomous-, multiple-answer- (MA), and open-field questions.

Part One, to be completed by a board representative, included the following topics:

- SGE characteristics: number and nature of therapeutic SGE (land-based, hydrotherapy or combination); number of participants and duration and frequency of sessions of therapeutic SGE; number and nature of other SGE (e.g., walking or running, Nordic Walking, Tai Chi).
- Organisational characteristics: responsibilities of associations (regarding organising and financing accommodation, equipment, supervision, or membership campaigns); funding sources (agreements with health insurances, membership dues, and sponsoring or funding from Dutch Arthritis Society); existence and frequency of structural and incidental evaluations of SGE contents, and organisation with members and supervisors (dichotomous questions).
- Recruitment of supervisors: nature of activities (advertising online, by the departing supervisor or through own network); selection criteria for supervisors (experience with guiding axSpA patients, experience with guiding exercise groups, membership local rheumatism network, or adequate education); perceived success in finding adequate supervision.
- Recruitment of members (patients): nature of activities (advertising in own media, in door-to-door magazines, through general practitioners, through physical therapists or through rheumatology clinics); developments in number of members over time (inclined, unchanged, or declined).
- Barriers: experienced barriers and challenges which the board member would like to see changed or improved (open-field question).

Part Two, to be completed by a supervisor of SGE (either axSpA-specific or for any RMD);

- Characteristics of supervisors: number of SGE supervisors per association and per group; professional background (physical therapist, physical therapy student or sports, and exercise instructor); years of experience with SGE (< 1 year, 1–5 years, > 5 years); completion of postgraduate education on RMD (yes/no); membership of a professional local rheumatology network (yes/no).
- SGE contents: therapy modalities used (joint mobility exercises, muscle strengthening exercises, aerobic exercise, breathing exercises, functional exercises, walking exercises, swimming exercises, relaxation exercises, volleyball or other sports, or passive mobilisation techniques). In addition, there were questions on the use of heart rate monitoring (yes/no), providing education on axSpA (yes/no), periodic assessments (yes/no; if so, frequency and measurement instruments used), and exercise personalization (individual goal setting and training schedule; yes/no).

### Statistical analysis

The analyses were done separately for axSpA-specific SGE and for SGE for people with any RMD. The categorical data are presented as frequencies and percentages and the continuous data as means with standard deviations or medians and range, where appropriate. The statistical analyses were performed using IBM SPSS Statistics for Windows, version 23.0 (IBM Corp., Armonk, NY, USA).

## Results

The questionnaire was returned by the board members of 67 of the 82 (82%) local patient associations. Forty-three (64%) of these 67 associations were involved in organising SGE for people with axSpA: 17/67 (25%) provided SGE specifically for axSpA and 26/67 (39%) for people with any RMD (not exclusively for axSpA). From all these 43 associations involved in organising SGE, a supervisor had completed survey Part Two on SGE contents and supervisor characteristics (*n* = 43). As shown in Table 1, axSpA-specific SGE (*n* = 17 associations) was offered to 56 groups, including a total of 684 participants, whereas SGE for any RMD (*n* = 19 associations; seven respondents did not provide these data) was offered to 167 groups, including 1940 participants.

**Table 1:** Characteristics of supervised group exercise (SGE) programmes for people with axSpA in the Netherlands

	axSpA-specific SGE (LPA *n* = 17)	SGE for any RMD (LPA *n* = 26)
Total number of groups, *n*	56	167 (LPA *n* = 19)^a^
Total number of participants, *n*	684	1940 (LPA *n* = 19)^a^
Combined land and hydrotherapy groups, *n* (%)	31/56 (55.4)	28/167 (16.8)
Number of LPA organising this, *n* (%)	16/17 (94.1)	12/26 (46.2)
Groups per LPA, Mdn (range)	2 (1–4)	1 (1–15)
Total number of participants, *n*	370	262 (LPA *n* = 19)^a^
Participants per LPA, Mdn (range)	20 (12–75)	18 (8–80)
Frequency, sessions per week, Mdn (range)	1 (1–1)	1 (1–1)
Duration, minutes per session, Mdn (range)	102.5 (45–180)	90 (60–150)
Land-based only therapy groups, *n* (%)	0/56 (0)	7/167 (4.2)
Number of LPA organising this, *n* (%)	n/a	7/26 (26.9)
Groups per LPA, Mdn (range)	n/a	1.5 (1–3)
Total number of participants, *n*	n/a	79 (LPA *n* = 19)^a^
Participants per LPA, Mdn (range)	n/a	16 (6–41)
Frequency, sessions per week, Mdn (range)	n/a	1 (1–1)
Duration, minutes per session, Mdn (range)	n/a	60 (45–90)
Hydrotherapy only groups, *n* (%)	13/56 (23.2)	120/167 (71.9)
Number of LPA organising this, *n* (%)	2/17 (11.8)	19/26 (73.1)
Groups per LPA, Mdn (range)	6.5 (3–10)	6 (1–19)
Total number of participants, *n*	168	1466 (LPA *n* = 19)^a^
Participants per LPA, Mdn (range)	84 (45–123)	80 (3–230)
Frequency, sessions per week, Mdn (range)	1 (1–1)	1 (1–3)
Duration, minutes per session, Mdn (range)	45 (30–60)	60 (30–60)
Other SGE^b^, *n* (%)	11/56 (19.6)	12/167 (7.2)
Number of LPA organising this, *n* (%)	4/17 (23.5)	7/26 (26.9)
Groups per LPA, Mdn (range)	2 (1–6)	5 (2–5)
Total number of participants, *n*	136	133 (LPA *n* = 19)^a^
Participants per LPA, Mdn (range)	24.5 (12–75)	40 (18–75)
Frequency, sessions per week, Mdn (range)	1 (1–1)	1 (1–1)
Duration, minutes per session, Mdn (range)	60 (60–90)	60 (60–90)

*AxSpA* axial spondyloarthritis, *RMD* rheumatic and musculoskeletal disease, *LPA* local patient association, *Mdn* median, *n/a* not applicable

^a^Seven respondents did not provide these data

^b^This refers to other forms of SGE with only sports, i.e., yoga, Tai Chi, volleyball classes, and Nordic Walking groups

### SGE characteristics and contents

As shown in Table 1, the SGE in the 17 local patient associations organising axSpA-specific SGE consisted most frequently of a programme combining land-based exercise and hydrotherapy (16/17, 94% of these associations). Two associations (12%) organised hydrotherapy only programmes, of which one also organised the combination programme. In addition to these therapeutic SGE programmes, four associations (24%) also organised other SGE, which included only sports, i.e., volleyball, Nordic Walking, or Tai Chi classes, specifically for people with axSpA.

With regard to SGE for any RMD, which was organised by 26 local patient associations, most associations organised a programme with only hydrotherapy (19/26, 73% of these associations), followed by the programme combining land-based exercise and hydrotherapy (12/26, 46% of associations). Furthermore, seven (27%) organised a programme with solely land-based therapeutic SGE and seven (27%) provided other SGE, which involved yoga or Tai Chi in four cases and Nordic Walking in the other three cases. Eleven associations (42%) provided more than one type of programme.

The frequency of SGE was once weekly for all associations besides one, where participants participated in the hydrotherapy three times weekly. The duration varied between the different types of programmes, with the combination programme taking the longest (mostly 90 min or more), the land-based SGE programmes having a median of 60 min, and the programmes with only hydrotherapy having a median duration of 45 min in the axSpA-specific SGE and 60 min in the SGE for any RMD.

As shown in Table 2, the most common exercise modalities in both axSpA-specific SGE and SGE for any RMD and on land as well as in water were joint mobility exercises and muscle strengthening (between 88 and 100%), whereas functional exercises were least popular (24% in axSpA-specific SGE and 39% in SGE for any RMD). Aerobic exercises were used by around half: on land by 59% and 61% of associations with axSpA-specific SGE and SGE for any RMD, respectively, and in water by 47% and 42%, respectively.

**Table 2:** Modalities of supervised group exercise (SGE) for people with axSpA in the Netherlands

	axSpA-specific SGE (total LPA *n* = 17)		SGE for any RMD (total LPA *n* = 26)	
	On land (*n* = 17)	In water (*n* = 17)	On land (*n* = 18)	In water (*n* = 24)
Therapy modalities, *n* (%)				
Joint mobility exercises	15/17 (88.2)	16/17 (94.1)	18/18 (100)	22/24 (91.7)
Muscle strengthening exercises	15/17 (88.2)	17/17 (100)	18/18 (100)	22/24 (91.7)
Aerobic exercises	10/17 (58.8)	8/17 (47.1)	11/18 (61.1)	10/24 (41.7)
Breathing exercises	11/17 (64.7)	6/17 (35.3)	11/18 (61.1)	14/24 (58.3)
Functional exercises	4/17 (23.5)	n/a	7/18 (38.9)	n/a
Walking exercises	13/17 (76.5)	16/17 (94.1)	8/18 (44.4)	20/24 (83.3)
Swimming exercises	n/a	14/17 (82.4)	n/a	15/24 (62.5)
Relaxation exercises	12/17 (70.6)	11/17 (64.7)	14/18 (77.8)	18/24 (75)
Sports	15/17 (88.2)	15/17 (88.2)	10/18 (55.6)	15/24 (62.5)
Volleyball	12/17 (70.6)	n/a	7/18 (38.9)	n/a
Passive mobilisation	6/17 (35.3)	5/17 (29.4)	7/18 (38.9)	7/24 (29.2)

*AxSpA* axial spondyloarthritis, *RMD* rheumatic and musculoskeletal disease, *LPA* local patient association, *n/a* not applicable

Table 3 shows that both in axSpA-specific SGE as well as with SGE for any RMD, the use of heart rate monitoring (6% and 4%, respectively), periodic assessments (12% and 23%), and personalisation according to assessments (6% and 19%) was relatively rare. If measurement instruments were used, the 6 min Walk Test (12% and 19%, respectively) and joint mobility tests (both 12%) were most often employed. Education on axSpA was provided in 47% of axSpA-specific SGE programmes and in 35% of SGE programmes for any RMD.

**Table 3:** Additional contents during supervised group exercise (SGE) for people with axSpA in the Netherlands

	axSpA-specific SGE (LPA *n* = 17)	SGE for any RMD (LPA *n* = 26)
Use of heart rate monitoring during exercise, *n* (%)	1 (5.9)	1 (3.8)
Providing education on axSpA, *n* (%)	8 (47.1)	9 (34.6)
Using periodic assessments, *n* (%)	2 (11.8)	6 (23.1)
Frequency assessments, times per year, Mdn (range)	1 (1–1)	2 (1–4)
Exercise personalisation according to assessments, *n* (%)	1 (5.9)	5 (19.2)

*AxSpA* axial spondyloarthritis, *RMD* rheumatic and musculoskeletal disease, *LPA* local patient association, *Mdn* median

### SGE supervisor characteristics

In all but one association, there was one supervisor guiding the exercise groups (in the other case, two supervisors guided one group). Table 4 presents the characteristics of the SGE supervisors. It shows that both in axSpA-specific SGE and in SGE for people with any RMD, most supervisors were physical therapists (92% and 71%, respectively) and had experience for more than 1 year in supervising SGE (90% and 84%, respectively). Furthermore, a minority of the supervisors had postgraduate education on RMD (36% and 20%, respectively) and were a member of a local rheumatology network (17% and 7%, respectively).

**Table 4:** Supervisor characteristics of supervised group exercise (SGE) for people with axSpA in the Netherlands

	axSpA-specific SGE (LPA *n* = 17)	SGE for any RMD (LPA *n* = 26)
Total number of supervisors, *n*	59	87
Number of supervisors per LPA, Mdn (range)	3.5 (1–11)	3 (1–13)
Number of supervisors per group, Mdn (range)	1 (1–2)	1 (1–1)
Professional background supervisors		
Physical therapist, *n* (%)	54/59 (91.5)	62/87 (71.3)
Physical therapy student, *n* (%)	3/59 (5.1)	4/87 (4.6)
Sports and exercise instructor, *n* (%)	2/59 (3.4)	21/87 (24.1)
Experience guiding group exercise		
< 1 year, *n* (%)	6/59 (10.2)	13/82 (15.9)
1–5 years, *n* (%)	18/59 (30.5)	40/82 (48.8)
> 5 years, *n* (%)	35/59 (59.3)	29/82 (35.4)
Postgraduate education on RMD, *n* (%)	21/59 (35.6)	16/87 (19.5)
Membership local rheumatology network, *n* (%)	10/59 (16.9)	6/87 (7.3)

*AxSpA* axial spondyloarthritis, *RMD* rheumatic and musculoskeletal disease, *LPA* local patient association, *Mdn* median

### Recruitment and selection of supervisors

As shown in Table 5, both for axSpA-specific SGE and SGE for any RMD, the most common procedures for recruitment of supervisors were through own networks of the associations (88% and 65%, respectively), followed by recruitment by the departing supervisor (47% and 54%, respectively). In both types of associations, the top three used selection criteria for supervisors were: experience with supervising exercise groups (65% and 69%, respectively), experience with supervising and/or treating people with axSpA (59% and 50%, respectively), and adequate postgraduate education (29% and 27%, respectively). The large majority of associations reported to be successful in finding supervisors meeting their criteria (94% and 77%, respectively).

**Table 5:** Recruitment characteristics of supervised group exercise (SGE) for people with axSpA in the Netherlands

	axSpA-specific SGE (LPA *n* = 17)	SGE for any RMD (LPA * n* = 26)
Procedures for recruitment of supervisors		
Advertising online (e.g., vacancy website), *n* (%)	0 (0)	2 (7.7)
By the departing supervisor, *n* (%)	8 (47.1)	14 (53.8)
Through own network, *n* (%)	15 (88.2)	17 (65.4)
No recruitment activities	1 (5.9)	7 (26.9)
Selection criteria for supervisors		
Experience with guiding axSpA patients, *n* (%)	10 (58.8)	13 (50)
Experience with guiding exercise groups, *n* (%)	11 (64.7)	18 (69.2)
Membership local rheumatology network, *n* (%)	1 (5.9)	5 (19.2)
Adequate education, *n* (%)	5 (29.4)	7 (26.9)
No selection criteria used, *n* (%)	3 (17.6)	2 (7.7)
Success in finding adequate supervision, *n* (%)	16 (94.1)	20 (76.9)
Membership campaign activities		
Advertising in own media, *n* (%)	8 (47.1)	21 (80.8)
Advertising in door-to-door magazines, *n* (%)	1 (5.9)	4 (15.4)
Advertising through general practitioners, *n* (%)	7 (41.2)	13 (50)
Advertising through physical therapists, *n* (%)	8 (47.1)	21 (80.8)
Advertising through rheumatology clinics, *n* (%)	17 (100)	24 (92.3)
Developments in number of members over time		
Increased, *n* (%)	1 (5.9)	10 (38.5)
Unchanged, *n* (%)	10 (58.8)	8 (30.8)
Decreased, *n* (%)	6 (35.3)	8 (30.8)

*AxSpA* axial spondyloarthritis, *RMD* rheumatic and musculoskeletal disease, *LPA* local patient association

### Recruitment of members

Concerning membership campaign activities (Table 5), the most frequently employed activities were similar for axSpA-specific SGE and SGE for any RMD: advertisement through rheumatology clinics was used by most associations (100% and 92%, respectively), followed by advertisement in own media and through physical therapists (both 47% and 81%, respectively). With regard to developments in number of members over time, Table 5 shows that just one of 17 (6%) associations with axSpA-specific SGE experienced an increase in memberships and six (35%) a decrease, whereas among the 26 associations with SGE for any RMD, ten (39%) reported an increase and eight (31%) a decrease.

### Organisational characteristics of SGE

As shown in Table 6, all local patient associations had financial responsibilities and all but three had organisational responsibilities (arranging accommodation or equipment or recruiting supervisors or members). Few associations had direct agreements with health insurance companies and in all but one association the funding sources included membership contributions as well as funding from the Dutch Arthritis Society, sometimes supplemented with commercial sponsoring. Respondents indicated that some patients were (partly) reimbursed for their membership contributions by their health insurance company.

**Table 6:** Organisational characteristics of supervised group exercise (SGE) for people with axSpA in the Netherlands

	axSpA-specific SGE (LPA *n* = 17)	SGE for any RMD (LPA *n* = 26)
Responsibilities of LPA regarding SGE		
Organising SGE accommodation, *n* (%)	15 (88.2)	14 (53.8)
Financing SGE accommodation, *n* (%)	16 (94.1)	24 (92.3)
Organising SGE equipment, *n* (%)	15 (88.2)	7 (26.9)
Financing SGE equipment, *n* (%)	16 (94.1)	10 (38.5)
Organising SGE supervision, *n* (%)	16 (94.1)	19 (73.1)
Financing SGE supervision, *n* (%)	15 (88.2)	23 (88.5)
Organising SGE membership campaigns, *n* (%)	15 (88.2)	23 (88.5)
No organisational SGE responsibilities, *n* (%)	1 (5.9)	2 (7.7)
No financial SGE responsibilities, *n* (%)	0 (0)	0 (0)
Agreements with health insurances^a^, *n* (%)	3 (17.6)	4 (15.4)
Funding sources		
Member contributions^a^, *n* (%)	17 (100)	25 (96.2)
Sponsoring, *n* (%)	5 (29.4)	6 (23.1)
Funding from Dutch Arthritis Society, *n* (%)	17 (100)	25 (96.2)
Evaluating group exercise with members		
Structurally, *n* (%)	12 (70.6)	13 (50)
Frequency, times per year, Mdn (range)	1 (1–4)	1 (1–9)
Incidentally, *n* (%)	1 (5.9)	9 (34.6)
No evaluation with members, *n* (%)	4 (23.5)	4 (15.4)
Evaluating group exercise with supervisors		
Structurally, *n* (%)	10 (58.8)	18 (69.2)
Frequency, times per year, Mdn (range)	1 (1–2)	2 (1–7)
Incidentally, *n* (%)	7 (41.2)	7 (26.9)

*AxSpA* axial spondyloarthritis, *RMD* rheumatic and musculoskeletal disease, *LPA* local patient association, *Mdn* median

^a^Some members declare their contributions with their health insurance themselves, which is sometimes (partly) reimbursed

With regard to the evaluation of the organisation of SGE, either structurally or incidentally, this was done with supervisors by all but one associations. However, evaluations among participating patients were done less frequent, namely by 24% and 27% of associations organising axSpA-specific SGE and SGE for any RMD, respectively.

A large majority of the board representatives reported barriers or aspects they would like to see improved, both among the associations with axSpA-specific SGE (88%) and associations with SGE for people with any RMD (81%). The most reported barrier was related to funding (41% and 27%, respectively), followed by finding new (or younger) members (41% and 8%, respectively). Other mentioned barriers concerned facilities, internal communication (by board with supervisors and members), finding new supervisors, and difference in exercise level within groups, but all these barriers were mentioned by two associations or fewer.

## Discussion

As a first step towards evidence-based revision of the practice of axSpA-specific SGE, this cross-sectional study examined the organisation and contents of SGE for people with axSpA in the Netherlands using a survey among local patient associations. It was found that 17 out of 67 associations responding to the survey offered axSpA-specific SGE, with most programmes combining land-based exercises, sports, and hydrotherapy. Most supervisors lacked postgraduate education on RMDs and the application of intensity monitoring, patient education, periodic assessments, and personalisation, needed for optimising the dosage in particular of aerobic exercise, was rare.

When compared with a similar Dutch cross-sectional study from 1994 [18], it appears that little has changed over the last two and a half decades. Current practice in The Netherlands also appears to be in line with relatively recent studies on the delivery of SGE for patients with axSpA from Switzerland and the United Kingdom [20–22]. In all, the focus is still mainly on joint mobility and muscle strengthening exercises, combined with sports and hydrotherapy, provided during once weekly sessions of relatively long duration.

These findings show that there is room for improvement, in particular regarding the provision of adequately dosed aerobic exercises [9–11, 16, 26]. Recent literature suggests that especially aerobic exercise with high intensity is beneficial for people with axSpA [16, 27]. To ascertain the execution of aerobic exercise with adequate intensity, implementation of intensity monitoring as well as a more personalised approach are needed, with every participant undergoing a comprehensive assessment, setting of individual goals, and periodic evaluations [11]. Furthermore, recent recommendations from the European League Against Rheumatism (EULAR) prescribe aerobic exercise to be performed with moderate or vigorous intensity on at least 5 or 3 days per week, respectively [8]. Once weekly SGE is thus not sufficient to achieve this frequency, so there should at least be education and personalised advice on additional exercise and physical activity acquired throughout the week. As patient education is currently only provided in less than half of the programmes, this element requires attention.

Although the data were collected in 2016, they constituted the basis for a pilot implementation project that was done in 2017 and 2018 in four regions (out of 17 regions providing axSpA-specific SGE and out of a total of 43 regions providing SGE for any RMD) [19], which has shown that it is highly likely that the situation has not changed since 2016 (apart from the four pilot regions). After all, from close contact with the Dutch Arthritis Society and many local patient associations during this project, there were no signs of any changes after 2016. Moreover, the pilot implementation showed to be hampered by various barriers. The SGE enhancements are therefore currently still warranted and a desired future action is to engage with local patient associations and other stakeholders to jointly examine how to cope with potential barriers during future implementations.

To implement the proposed SGE enhancements, the supervisors require adequate education. However, similar to a few decades ago [18], the large majority of SGE supervisors in this study consisted of physical therapists without a postgraduate education on RMDs. Physical therapists’ knowledge on RMDs is an important facilitator for the implementation of high-intensity aerobic exercise [21] and is one of the core competencies of health professionals in rheumatology [28]. Improving the knowledge of physical therapists could be hampered by the limited availability of postgraduate education on RMDs in many European countries, in particular with a specific focus on exercise and axSpA [29]. A number of (online) courses for health professionals addressing axSpA in English are available, such as those developed by the EULAR [30] or by the National Axial Spondyloarthritis Association (NASS) from the United Kingdom [31]. However, lack of English language skills could be a barrier for participation among health professionals in many non-English speaking countries [29]. Fortunately, in the Netherlands, recommendations for physical therapists on exercise and axSpA recently became available [32] as well as a course on implementing these recommendations [33].

In addition to availability of appropriate education, it is also important that SGE supervisors are motivated to participate in such courses and that patient associations use postgraduate education on RMDs as a selection criterium when recruiting supervisors. In this study, only a minority of the supervisors had postgraduate education and less than 30% of associations used it as a selection criterium. However, it is likely that patient associations currently limit the demands on their supervisors, because the payment for supervising SGE is probably lower than for regular therapy (personal communication). Limited funding could be the main obstacle, since funding was the SGE barrier mentioned most often by the boards of the patient associations. Funding mainly exists of contributions from members themselves and from the Dutch Arthritis Society. Only a few associations have direct agreements with health insurances. When implementing the SGE enhancements, this barrier should be taken into account and suitable payment of SGE supervisors should be provided.

When implementing the suggested SGE enhancements, the patients’ perspective should be accounted for. One study, examining the perspective of axSpA-specific SGE participants towards current SGE and the proposed SGE enhancements, found that the majority of axSpA patients was satisfied with the current axSpA-specific SGE, but also agreed with intensity monitoring, periodic assessments, and exercising more frequently [19]. Half of the participants were in favour of education and a large majority found specialised supervision highly important. However, that study also showed that the majority of SGE participants in the Netherlands had a relatively high age and participated in SGE for a long time [19]. This is in line with the current finding that recruiting new and younger SGE members is a challenge mentioned most often (besides funding) by the associations’ boards. To attract younger axSpA patients, it may help to implement more education on self-management, as was found in one study using focus groups [34], or by exploring and using technological possibilities such as web-based home exercise programmes [35], which provide more flexibility in exercise times, costs and distance than traditional SGE sessions [36]. Furthermore, a study among axSpA patients registered in a hospital in the United Kingdom found that over half of them were not familiar with the NASS [37]. To recruit more (young) SGE members, it might help to increase awareness regarding associations organising axSpA-specific SGE.

There are three study limitations to be mentioned. First, using a non-validated survey, the data could be affected by various forms of bias, including social desirability bias regarding the contents of SGE. To limit the risk of this bias, it was made clear to participants that the survey was meant to make an inventory of the current SGE situation and to assess the needs to improve it, rather than to make a judgement of the quality of the SGE they provided. Moreover, as this study included a survey among both board members and the SGE supervisors, it is expected that the combined responses provide a realistic picture of the situation. Second, there were no specific questions in the survey about providing home exercise advice. Advice on home exercise is important for achieving adequate exercise frequency and findings from another study suggest that it is currently lacking in axSpA-specific SGE [19]. Finally, this study only examined SGE organised by local patient associations affiliated with the Dutch Arthritis Society and axSpA-specific SGE organised outside of these associations were not included. However, it seems unlikely that axSpA-specific SGE exists beyond these associations in the Netherlands. As this study showed a high response rate among the local patient associations (82%), the results may be well generalisable to all axSpA-specific SGE in the Netherlands.

In conclusion, most SGE programmes for patients with axSpA in the Netherlands contained a combination of land-based exercises and hydrotherapy, with the main focus on joint mobility, muscle strength, and sports. To meet current scientific insights, there should be more focus on adequately dosed aerobic exercises, by implementing intensity monitoring, patient education, periodic assessments, and exercise personalisation, and by providing and promoting postgraduate education for supervisors.

## Data Availability

The data underlying this article will be shared on reasonable request to the corresponding author. The data were presented with two posters at the Annual European Congress of Rheumatology (EULAR 2017) and abstracts have, therefore, been published: 1. Giesen, F., Van Weely, S., Lopuhaä, N., Vliet Vlieland, T. (2017). THU0730-HPR Content and supervision of group exercise therapy (GET) for axial spondyloarthritis (axSpA) in the Netherlands; a nationwide survey. *Annals of the Rheumatic Diseases*, 76, 1479.2-1479. 2. Giesen, F., Van Weely, S., Lopuhaä, N., Vliet Vlieland, T. (2017). PARE0022 Organisation of group exercise therapy (GET) for patients with axial spondyloartritis (axSpA) in the Netherlands; a nationwide survey. *Annals of the Rheumatic Diseases*, 76, 1559.3-1559.
